# In-Site and Ex-Site Date Palm Exposure to Heavy Metals Involved Infra-Individual Biomarkers Upregulation

**DOI:** 10.3390/plants10010137

**Published:** 2021-01-12

**Authors:** Zayneb Chaâbene, Agnieszka Rorat, Walid Kriaa, Imen Rekik, Hafedh Mejdoub, Franck Vandenbulcke, Amine Elleuch

**Affiliations:** 1 Laboratory of Plant Biotechnology, Faculty of Sciences of Sfax, University of Sfax, Sfax 3000, Tunisia; hafedh.mejdoub@fss.rnu.tn (H.M.); amineelleuch@hotmail.com (A.E.); 2 Laboratoire de Génie Civil et géo-Environnement–Université de Lille 1, F-59655 Villeneuve d’Ascq, France; agnieszkarorat@gmail.com (A.R.); franck.Vandenbulcke@univ-lille.fr (F.V.); 3 Environmental Science Center, Qatar University, Doha P.O. Box 2713, Qatar; maswkri@yahoo.fr; 4 High Institute of Applied Biology of Medenine, Medenine 4119, Tunisia; imenbmc@yahoo.fr

**Keywords:** atmospheric contamination, gene expression, metallic stress, integrative biomarkers, metal accumulation

## Abstract

As a tree of considerable importance in arid regions—date palm, *Phoenix dactylifera* L. survival in contaminated areas of Sfax city has drawn our attention. Leaf samples of the plants grown in the study area showed high levels of cadmium (Cd), copper (Cu), and chromium (Cr). On the basis of this finding, the cellular mechanisms that explain these metal accumulations were investigated in controlled conditions. After four months of exposure to Cd, Cr, or Cu, high bioconcentration and translocation factor (TF > 1) have been shown for date palm plantlets exposed to Cd and low TF values were obtained for plantlets treated with Cr and Cu. Moreover, accumulation of oxidants and antioxidant enzyme activities occurred in exposed roots to Cu and Cd. Secondary metabolites, such as polyphenols and flavonoids, were enhanced in plants exposed at low metal concentrations and declined thereafter. Accumulation of flavonoids in cells may be correlated with the expression of the gene encoding Pdmate5, responsible for the transport of secondary metabolites, especially flavonoids. Other transporter genes responded positively to metal incorporation, especially *Pdhma2,* but also *Pdabcc* and *Pdnramp6*. The latter would be a new candidate gene sensitive to metallic stress in plants. Expressions of gene coding metal chelators were also investigated. *Pdpcs1* and *Pdmt3* exhibited a strong induction in plants exposed to Cr. These modifications of the expression of some biochemical and molecular based-markers in date palm helped to better understand the ability of the plant to tolerate metals. They could be useful in assessing heavy metal contaminations in polluted soils and may improve accumulation capacity of other plants.

## 1. Introduction

Based on their physicochemical properties, bioactive-metals are divided into two groups: the redox-active metals, such as chromium (Cr), copper (Cu), and iron (Fe), and redox-inactive metals, such as cadmium (Cd), nickel (Ni), aluminum (Al), and zinc (Zn). The metals belonging to the first group are included in a fundamental bioprocess and play a pivotal role in oxygen formation, and enzyme and protein structure [1]. They can directly generate oxidative injury via undergoing Haber–Weiss and Fenton reactions, which leads to an uncontrolled formation of reactive oxygen species (ROS) in plants. At high levels, they may cause cell homeostasis disruption, defragmentation of biological macromolecules, such as DNA (DNA strand breakage), proteins and lipids, or cell membrane and damage of photosynthetic pigments, which may trigger cell death [2]. Via inhibiting antioxidative enzymes, inducing ROS-producing enzymes (NADPH oxidases) and glutathione depletion, non-essential elements that belong to the redox-inactive metals group can indirectly inflict oxidative stress in living organisms [3]. Yet, in numerous cases, such as cadmium stress in liverwort *Conocephalum conicum* (Marchantiales; [4]), the metal induces the activity of antioxidant enzymes rather than inhibits it.

Despite its redox state, the concentration of both essential and non-essential metals is crucial for environment. They become toxic to living organisms above a critical concentration. Even those classified as essential can be toxic if present in excess [5].

A slightly elevated concentration of Cu can induce phytotoxicity [6], i.e., affecting plant growth and altering cellular antioxidant system [7]. The pigment and protein components of photosynthetic membranes are the targets of Cu ions, inducing perturbation of photosynthetic metabolisms [8]. Thereby, in presence of high Cu amounts, leaves suffer chlorosis and necrosis caused by the inhibition of chlorophyll and carotenoid biosynthesis, which delays the incorporation of these pigments into photosystem complexes. Cu may also reduce absorption of essential nutrients, especially Fe [9]. A non-essential metal, such as Cd, negatively affects plant growth and development, and even causes plant death. Hazrat et al. [10] characterized Cd as an extremely worrying pollutant due to its high toxicity and large solubility in water. It is also recognized as one of the most phytotoxic heavy metal (HM) contaminants [10]. Its toxicity is correlated to the alteration of the uptake and distribution of macro- and micro-nutriments, especially Fe and Magnesium (Mg) in plants [11]. Moreover, chlorophyll is one of its targets and chlorosis may due to Fe deficiency caused by root Fe(III) reductase inhibition [12], substitution of central Mg ion in the chlorophyll molecules [13], and/or by enhancement of chlorophyll catabolic activity, which may seriously affect photosynthesis [14]. Cr, the seventh most abundant metal on earth, is considered as one of the most dangerous toxic HM to living organisms and ecosystems due to its wide industrial applications [15]. Being a strong oxidizer, Cr (VI), the second stable form of Cr, is highly toxic and more mobile in soil/water systems, even at low concentrations [15]. Cr phytotoxicity can result in reduction of root growth and biomass, perturbation of nutrient balance and enzyme activities, degradation of pigment status and induction of leaf chlorosis, and oxidative stress in plants [16].

Plants, especially tree species with their long reproductive cycles, have evolved a complex network of highly effective homeostatic mechanisms. They serve to maintain physiological concentrations of essential metal ions, such as Cu, and minimize exposure to non-essential metals, such as Cd and Cr [17]. Exploring the mechanisms at infra-individual levels, using transcriptomic, proteomic, metabolomic approaches allow insight into the physiology, the biochemistry, or the cell biology of exposed organisms, and sometimes the identification of exposure biomarkers. Some mechanisms, usually described as ubiquitous, are required to minimize the damage caused by high concentrations of elements [18]. Plant cells contain well-equipped antioxidative defense elements, activated by HMs and aim to attain a new balanced redox status. This signal transduction network involves stress-related proteins, such as ROS-removing enzymes and non-enzymatic antioxidants, including low molecular mass antioxidants scavengers [3]. Other mechanisms target individual metal ions to control the accumulation, trafficking, and detoxification of metals. The latter are devoted to metals and they may occur at transcriptional level. The transcriptional expression of specific metal-responsive genes (to counteract the stress stimuli) are described as infra-individual biomarkers or molecular biomarkers [19]. When the metal comes into the plant, the plant cell activates specific genes, such as *pcs* [20], which control the production of chelating compounds phytochelatin (PCs; [21,22]). Metallothioneins (MTs) are able to bind metal ions and are produced from mRNA translation. The overexpression of *mt* to increasing concentrations of HMs was observed in plants [23]. In addition, the plasma membrane transporters in plants participate in HMs stress responses, as they are involved in metal uptake and homeostasis. Numerous families of transporter genes were identified. The plant ATP-binding cassette (ABC) family plays an important role in the general detoxification mechanism. The *abc* expressions are enhanced by xenobiotic incorporation [24]. PCs and PC-HM complexes are transported by ABC C subfamily-type transporters [25]. P-type ATPase was reported to be involved in nutrient uptake and distribution [26]. Among them, the transporters belong to the heavy metal P_1B_-ATPase subfamily, including HM-transporting P-type ATPase (HMA; [27]) and the Natural resistance-associated macrophage protein (NRAMP) transporters [28]. The Multidrug and toxic compound extrusion (MATE) proteins are involved in extrusion of multidrug and toxic compound from the cell.

It has been shown that date palm (*Phoenix dactylifera*) has great capacity to remove HMs from wastewater [29] and is able to accumulate Cd and Cr [30]. Therefore, although plant detoxification mechanisms are extensively reported in literature, little is known about date palm responses to metals stress. The species is widely cultivated in the southern part of Tunisia country, where soils around opencast mines and near the industrial sites, especially the phosphate fertilizer industry exhibit very high contents of HMs [31,32]. In fact, erosion of phosphogypsum (PG) piles, waste of the treatment of phosphate rock (PR) containing highly polluting hazardous element, can cause the contamination of the surrounding areas [33].

The aims of the present work were to (i) monitor the level of accumulation of HMs in field-exposed date palm; (ii) estimate the accumulation of several major contaminants (Cd, Cu, and Cr) in various plant compartments in controlled conditions; (iii) explore the plant metal detoxification mechanisms involved in accumulation or avoidance of metals; and (iv) test the use of the studied infra-individual markers as biomarkers of metallic stress.

## 2. Material and Methods

### 2.1. Soils and Plants Sampling from the Study Site

**Figure 1:**
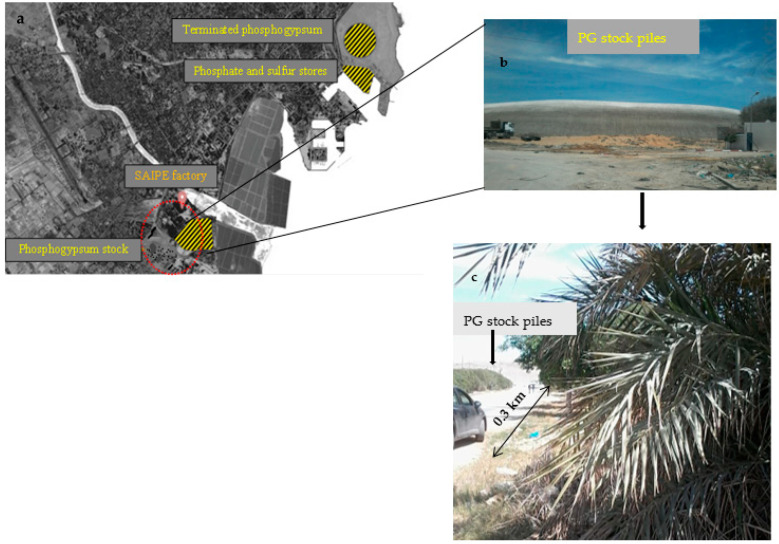
Show study area (**a**) in Sfax region (34°44′52.249″ N 10°45′58.187″ E), southern Tunisia (237 km), were the phosphogypsum (PG) stockpiles of the Industrial Society of Phosphoric Acid and Fertilizers (ISPAF) factory (**b**). Soil and plant sample collections from about 0.3 km far away from PG stockpiles (**c**).

The studied field was located in Sfax (34°44′52.249″ N 10°45′58.187″ E), southern Tunisia (237 km). It was about 0.3 km far away from the PG stockpiles of the Industrial Society of Phosphoric Acid and Fertilizers (ISPAF) factory and lead melting industry (Figure 1), the main source of HMs in Sfax [34]. Beyond this distance, the land becomes occupied by industrial constructions, streets, and houses. ISPAF emitted dust was estimated annually to be around 1610 tons [33], generating two piles of PG, of more than 15 million tons [34].

More than 20 soil samples were collected from different locations in the distance of 0.3 km from the PG piles identified using the Google Maps Distance Calculator (Figure 1). The samples were collected in a depth of surface soils of all sites (0–20 cm). Soil samples were homogenized, collected in plastic bags, brought to laboratory, air-dried, and stored for analysis. The date palm leaves samples were collected from the same sites as the soil samples. Leaves were washed with distilled water to remove soil particles, ground with liquid nitrogen, and stored at −20 °C for molecular analysis. Part of collected leaf samples were lyophilized and grounded in liquid nitrogen for biochemical analysis in three replicates.

### 2.2. Ex-Situ Soil Contamination and Plant Transfer

A mixture of 2/3 peat and 1/3 potting soil was prepared, dehydrated at 50 °C, and distributed in pots; 1 kg per box. Amount of 10, 50, and 100 mg of CdCl_2_ and of 50, 100, and 500 of CuSO_4_ and of K_2_CrO_4_ powder were homogenized in distilled water. After a complete dilution, dried soils were gradually soaked with the solution by long mixing.

Seedlings of date palm of about 2 months of germination in in vitro controlled conditions produced roots between 5 and 10 cm lengths. They were transferred to previously prepared pots, 4 plantlets per pot. The seedlings were grown in a greenhouse at 25 ± 3 °C and 16 h of photoperiod. When the plants were growing, they were sprayed with distilled water. In order to minimize leaching, the infiltrated irrigated water was recovered and reused again for irrigation. The pots were prepared in triplicates. After 4 months of growth, plants were harvested. Shoots and roots were separated and rinsed with distilled water, to ensure that outside contamination was removed. The fresh plant materials were ground in liquid nitrogen for chemical and molecular analysis. Plant tissues were oven-dried at 60 °C for 72 h and used for biochemical analysis. The analyses were done in triplicates.

### 2.3. Soil and Plant Digestion for Metal Spectroscopic Analysis and Data Processing

Soil and plant dried samples were lyophilized before plant material was ground to a fine powder with liquid nitrogen. One mL of HNO_3_ (65%, 108 m/V, trace pure) was added to 0.1 g of soil and plant tissue samples. The mixture was left at least 12 h at room temperature under a hood. Then, the mixture was heated to reflux for 2 h in a sand bath in 120 °C. Before a second digestion at 180 °C, 1 mL of acid mixture (HNO_3_:H_2_SO_4_:HClO_4_: 10:2:3, *v*/*v*/*v*) was added. The digests prepared in this way were then analyzed for the elements contents spectroscopically using atomic emission spectrometry with inductively coupled plasma, ICP–AES (Varian 720-ES, USA). Five elements were monitored in different compartments (soil, roots, and leaves): Cd, Cu, Cr, Mn, and Zn.

The bioaccumulation factor (BAF) defined by [35] was calculated as the ratio of metal concentration in the entire plant to that in the soil [35], and is given in Equation (1).

BAF = (Metal) plant/(Metal) soil(1)

The Translocation factor (TF) was described as the ratio of concentration of HM in plant shoot to that in plant root [35] and is given in Equation (2).

TF = (Metal) shoot/(Metal) root(2)

The Bioconcentration factor (BCF) was calculated as ratio plant roots HM to that of soil [36] and is given in Equation (3).

BCF = (Metal) root/(Metal) soil(3)

The enrichment factor (EF) was calculated as the ratio of metal concentration in above ground plant parts over metal concentration in soil and given in Equation (4). The EF is considered ideal HM stabilizers.

EF = (((Metal) stressed plant roots + leaves/(Zn) stressed plant roots + leaves)/((Metal) doped soil/(Zn) doped soil]/control)(4)

Zn is used as reference element.

### 2.4. Biochemical Analysis

Hydrogen peroxide concentration was determined, as previously described by Elleuch et al. [37]. The levels of products of lipid peroxidation were measured as thiobarbituric acid reactive substances (TBARS) aldehydes, according to Rustérucci et al. [38]. The concentrations of TBARS were calculated using an extinction coefficient of 155 mM^−1^ cm^−1^.

Total protein extraction from date palm roots and leaves was made according to Gómez-Vidal et al. [39]. Total protein content was determined spectrophotometrically according to the method of Bradford [40], using bovine serum albumin as a standard. Assays of antioxidant enzyme activities in date palm tissues were prepared for catalase (CAT), ascorbate peroxidase (APX), and superoxide dismutase (SOD) as described previously by Chaâbene et al. [41]. SOD activity was assayed using the photochemical nitro blue tetrazolium (NBT) method and measured according to Beyer and Fridovich [42]. CAT and APX activities were determined, as described by Aebi [43] and Nakano and Asada [44] methods, respectively.

The secondary metabolites were extracted by maceration of 50 mg of tissues powder in 2 mL of organic solvent (80% acetone), under ultrasonic conditions for 45 min, at 4 °C. After centrifugation, the supernatant containing phenols was recovered. A second identical extraction was carried out to extract 30% of additional phenols to obtain a more complete dosage. The supernatants were combined before being concentrated to dryness under vacuum. The total phenols content in tissue was determined using the Folin–Ciocalteu method, described by Pinelo et al. [45]. Total flavonoids were determined according to Zhishen et al. [46]. Based on the condensation of polyphenolic compounds with vanillin in an acid medium [47], the content of tannins in roots and leaves of date palm plantlets was measured.

### 2.5. Real Time qPCR Amplification

RNA extraction and cDNA synthesis were performed, as previously described by Chaâbene et al. [25]. RNAs were isolated with the Plant RNeasy mini kit (Qiagen, Courtaboeuf, France), including the on-column DNase digestion step. Concentration and purity of the RNA samples was determined using a Spectrophotometer (SPEC-TROstar Nano Microplate Reader). Reverse transcription was performed on 1 mg of total RNA from each sample using the random hexamer primers and the Maxima H Minus First strand (Thermo Scientific, USA) cDNA Synthesis Kit, according to the manufacturer’s instructions. Primer sequences of candidate genes were designed from the conserved domain found using Primer3Plus (http://frodo.wi.mit.edu/) and verified using NetPrimer and BeaconDesigner [25].

Real-time polymerase chain reaction (qPCR) amplification method, using MESA Blue qPCR Master Mix reagent kit (Eurogentec, Seraing, Belgium), was performed on reverse transcribed RNAs extracted from *Phoenix dactylifera* according to Brulle et al. [48]. The specific phytochelatin synthase type 1 (*Pdpcs1*) and metallothionein type 3 (*Pdmt3*), *Pdabcc family*, *Pdhma2*, *Pdmate5,* and *Pdnramp6* specific primers were used [30]. qPCR reactions were performed with a LightCycler 480 Real Time PCR system (RocheDiagnostics, Mannheim, Germany), according to previously described procedures [43]. Real-time PCR efficiencies (E) were calculated from the given slope of the standard curve, according to equation E = 10^(−1/slope)^. E values ranged from 1.91 to 2 (with 100% = 2) and calculated from a standard curve. The expression levels and the relative fold expression (RFE) were determined, according to previously described procedures [49]. The geometric mean of the three most stable reference genes in control and Cd, Cr, and Cu-stressful conditions identified by Chaâbene et al. [30,41] was used to calculate expression of target gene levels, according to Brulle et al. [48]. Absolute quantification of genes expression levels is shown as log_2_. *Pdpcs1* and *Pdmt3* relative expression levels were normalized to those of the reference genes selected after gene expression validation [25].

### 2.6. Statistical Analysis

Comparative threshold values represent the mean of three repetitions of the same sample. Results were expressed as means ± SD. All analyses were conducted using STATISTICA 10. Significant differences between parameters were tested using Tukey’s HSD test after one-way and two-way ANOVA, with the type of tissues and metal concentrations as the two factors. Differences at *p* < 0.05 were considered statistically significant.

## 3. Results

### 3.1. Soil Contamination and Metal Concentrations in Field

Soil collected in the contaminated area of Sfax region (Figure 1), showed Cr concentration of 28 mg kg^−1^ dry soil. Such concentration, as well as Cu level in field soil samples, were within permissible limits recommended by Dutch standard [50], for instance 100 mg kg^−1^ for Cr and 36 mg kg^−1^ for Cu. Cd concentration was 0.9 mg kg^−1^ (Table 1) which slightly exceed Dutch standard permissible limit (0.8 mg kg^−1^). This was the case in the vicinity of PG stockpiles in Lebanon, where the environment was found to be contaminated with HMs attending toxic amounts considerably above-threshold of Cd, Zn, and radionuclide (U; [51]). However, Cd and Cu levels in harvested leaves of date palm in contaminated fields were almost two times higher than in the soil (Table 1). All tested metals in plant leaves exceeded the metal common range, according to the World Health Organization (WHO) [52]. Cd, Cu, and Cr concentrations in date palm leaves from the contaminated site of the Sfax region were 1.6, 14.6, and 17.9 mg kg^−1^, respectively. The standard limits of WHO [52] are 0.02, 10, and 1.3 mg kg^−1^ for Cd, Cu, and Cr, respectively.

### 3.2. Metal Concentrations in Plants

Significant differences in metal concentrations were observed in various compartments (soil, roots, and leaves) compared to control plants (Table 1). After 4 months of exposure to Cd-contaminated soil, a significant bioaccumulation (i.e., BCF > 1) was observed in roots of plantlets growing in 10 and 50 mg kg^−1^ of Cd-spiked soil (Table 2). Moreover, an important Cd-translocation reaction of date palm was evidenced by a TF > 1 in samples treated with the lowest metal concentration (Table 2). It is noteworthy that Cd negatively influenced Mn uptake by date palm (Table 3). Significantly non-linear relationships between Cd and Mn was confirmed by the pairwise correlation coefficient (*r* = −0.61), implying the existence of an antagonistic effect of Cd on Mn absorption and translocation. Similarly, Cr^VI^ altered Mn translocation to date palm young leaves (Table 3). In addition, low TF under Cr-stress was found (Table 2). Cu concentration in roots ranges from 17.2 to 19.6 mg kg^−1^ (Table 1). BCF exceeding 1.3 and TF of 0.9 was observed for the lowest metal concentrations.

### 3.3. Accumulation of Oxidants in Plant under HMs Stress

In natural conditions, date palm plantlets generated H_2_O_2_ within plant cells. The accumulation of hydrogen peroxide (H_2_O_2_) and the product of lipid peroxidation, in the form of TBARS significantly differ between tissues (*p* < 0.05). Leaves of the control plants accumulated oxidants more than roots (Table 4). Two-way ANOVA indicated that, except for Cr, stress caused by high concentrations of metals (Cd and Cu) in plant tissues influence H_2_O_2_ production in cells. Higher Cd amounts in soil further enhanced H_2_O_2_ and TBARS accumulation, especially in roots. However, no significant difference between roots and leaves exposed to Cu stress in case of H_2_O_2_ accumulation was shown (Table 4). The highest concentration of oxidant (H_2_O_2_ = 709.56 µmol/g fresh weight (FW) was noted in roots of young plantlets of date palm grown in 100 mg kg^−1^ of Cd amended soils whereas, the lowest (H_2_O_2_ = 317.79 µmol/g FW) was accrued in roots of plants exposed to 100 mg kg^−1^ Cr spiked soil. In roots exposed to 500 mg kg^−1^ of Cu or Cr, H_2_O_2_ amount in cells never exceeded 600 µmol/g FW (Table 4). Similarly, TBARS production, a biomarker of oxidative damage in cells, followed the same trend as H_2_O_2_ accumulation, i.e., it differed significantly between roots and leaves in Cd-stressed plants. No significant difference between tissues was shown for plants exposed to Cu or Cr treatment (Table 4).

### 3.4. Enzymatic Antioxidant and Secondary Metabolites Potential in Date Palm under HMs Stress

High levels of antioxidant enzyme activities were detected for all tested HMs (Table 4). In fact, except for date palm plantlets treated with Cr, CAT, APX, and SOD enzymes amounts were significantly much higher in leaves than in roots. CAT activity enhanced under Cd stress and reached its maximum at 10 mg kg^−1^ with 2.76 and 3.5 µmol H_2_O_2_/mg protein in roots and leaves respectively. Important amounts of Cu and Cr in soils (100 mg kg^−1^) stimulate CAT activity more than Cd (Table 4). CAT activity continued to increase even under 500 mg kg^−1^ of Cu or Cr concentrations, while it decreased under enhancing Cd stress. APX activity showed correlation with CAT activity. It was increased when CAT activity was reduced under 50 mg kg^−1^ Cd (Table 4). Indeed, APX activity was more prominent in Cd and Cr treated plantlets showing no significant differences between plants tissues treated with Cr (Table 4). Positive correlations were found between SOD activity and Cu and Cr concentrations in the roots and leaves of date palm young plants. The secondary plant metabolism was influenced by metal stress and plant tissues (*p* < 0.05) especially in samples treated with Cd (Table 4). Leaves accumulated more polyphenols, flavonoids, and tannins than roots. At low Cd stress (10 mg kg^−1^), flavonoids were more significantly induced compared to polyphenols and tannins (Table 4), while, no significant difference between tissues was found. Increasing Cd stress decreased production of non-enzymatic antioxidant metabolites. A harmful decline of tannins by more than 40% was observed in roots of plants treated with 100 mg kg^−1^ Cd. However, at high concentrations of Cr, tannins content was higher than in control. Flavonoid percentage was induced in roots under cupric stress (Table 4). Contrarily, roots of plants treated with Cr showed no significant decline of flavonoid content, which was less influenced by this metal than by Cd and Cu (Table 4). Polyphenol content declined only in roots and leaves of plants grown in soil containing 100 mg kg^−1^ Cd (Table 1). According to two-way ANOVA, polyphenol levels was influenced by the type of tissues and concentration of Cd and Cr. Thereby, non-linear relationships between the two factors was shown in young date palm plant exposed to cupric stress.

### 3.5. Profiling of HMs Related Gene Transcripts in Date Palm

**Figure 2:**
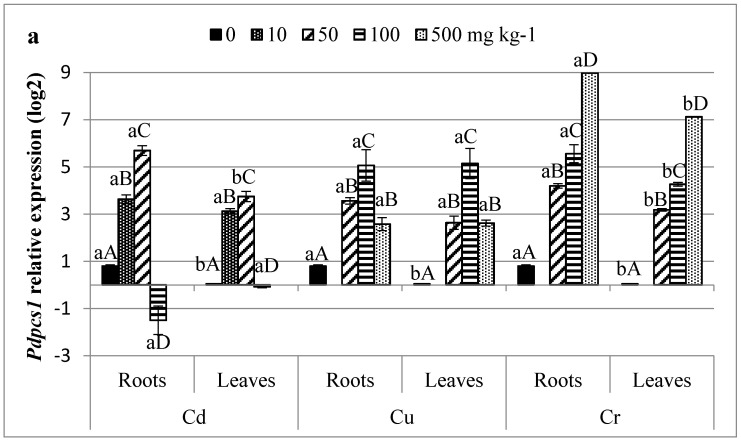

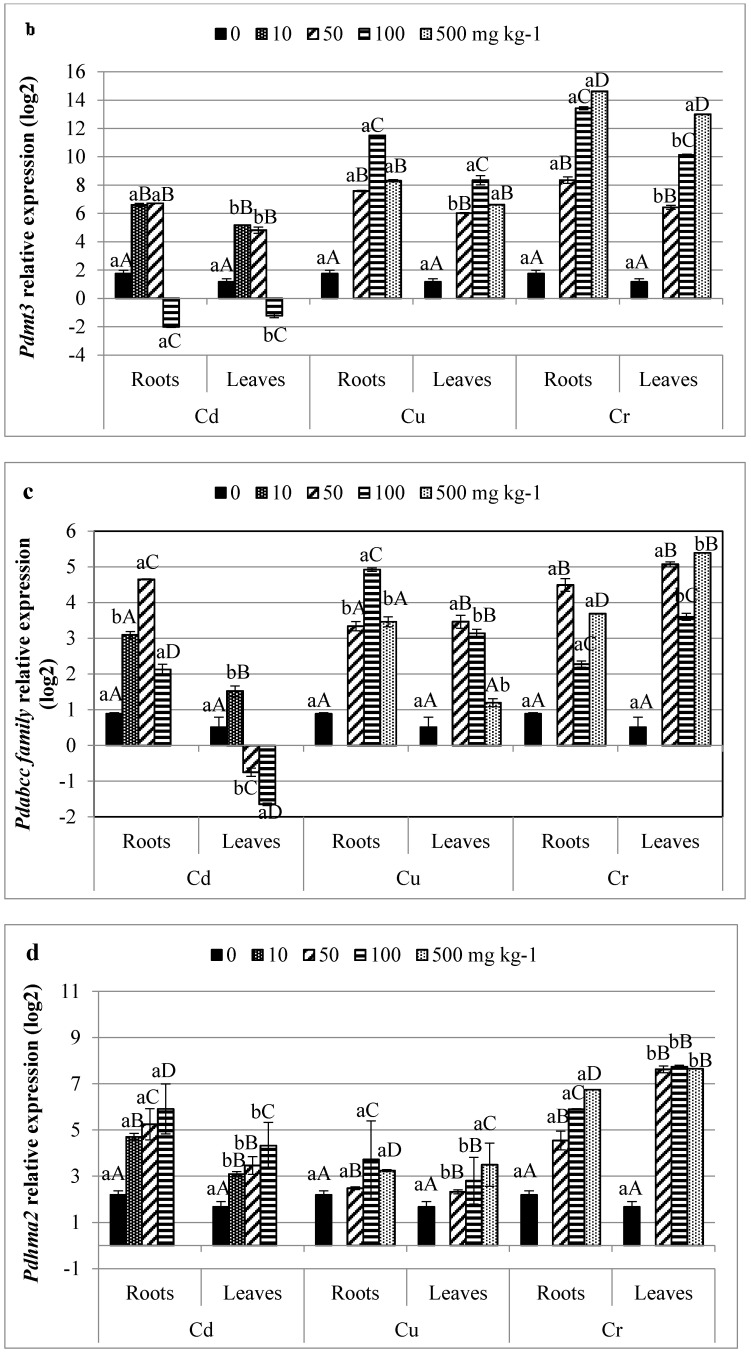

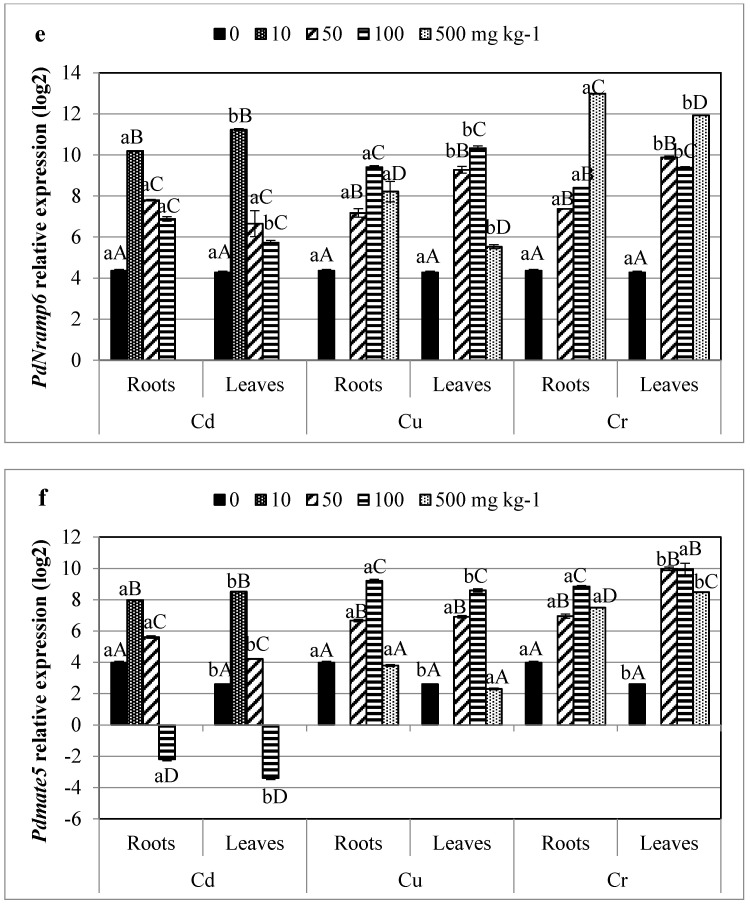
Relative expression factor of *Pdpcs1* (**a**), *Pdmt3* (**b**), *Pdabcc* (**c**), *Pdhma2* (**d**), *PdNramp6* (**e**), and *Pdmate5* (**f**) exposed to Cd, Cu, and Cr. Data presented are means ± standard error of three independent experiments. Differences between groups are shown as results of one-way ANOVA post-hoc Tukey’s test; where small letters show differences between tissues and major letters show differences between metal concentrations. Means not showing the same letter are statically different.

qPCR was performed for six genes; *Pdpcs1*, *Pdmt3*, *Pdabcc family*, *Pdhma2*, *PdNramp6,* and *Pdmate5*; in roots and leaves of date palm young plantlets grown during two months in –Cd, or –Cu, or –Cr spiked soils. The expression level of genes did not significantly differ between tissues of non-treated plants except for *Pdpcs1* and *Pdmate5*, which were predominantly expressed in roots (Figure 2). However, metal contaminations strongly influenced the expression of all tested genes (*p* < 0.05; Figure 2) and differences were observed between tissues. For instance, the highest relative expression level (14.61) was observed for *Pdmt3* in leaves exposed to high level of Cr (Figure 2b). Whereas, the highest expression factors of more than 80-, 110-, and 150-fold (data not shown) were obtained for *Pdpcs1* in leaves of plants exposed to 50 mg kg^−1^ Cd, 100 mg kg^−1^ Cu, and 500 mg kg^−1^ Cr, respectively. *Pdpcs1* transcript was mostly expressed in plantlets exposed at moderate concentration of Cd, while it decreased at 100 mg kg^−1^ Cd. Gene downregulations were also observed for *Pdmt3* and *Pdmate5* in roots and leaves of plants exposed to high doses of Cd (Figure 2b,f). Only Cd exposure caused downregulation of several genes. Downregulations were not observed in date palm exposed to Cu and Cr. *Pdhma2* was overexpressed in roots even at high Cd stress (Figure 2d). The latter gene was more induced by Cr^VI^ ions. Its expression increased with increasing Cu-stress in contrast to the other tested genes which expression decreased at 500 mg kg^−1^ Cu (Figure 2). Gene expression patterns of MATE5 gene significantly varied between tissues (Figure 2f). Indeed, it was downregulated at high Cd concentration especially in leaves. However, *Pdmate5* expression pattern was induced in Cr-treated leaves by almost 4-fold. The *Nramp* metal transporter gene expression has also been monitored (Figure 2e). In the present study, Cd^2+^, Cu^2+^, and Cr^6+^ ions induced *PdNramp6* expression mainly in plant roots. Cr was the most effective *PdNramp6* expression inductor.

## 4. Discussion

### 4.1. Metals Concentration and Their Interactions with Plant Nutrients

Considered as the most industrialized city of Tunisia, the southern edge of Sfax city suffers for long time ago by emission of particles and uncontrolled waste storage, such as PG piles of ISPAF factory. It was found that the latter released around 4.5 t/day of particulate matter containing sulfate, phosphorous compounds, and HMs in concentrations largely exceeding the permissible emission standards [53]. Arid climate of Sfax and especially low annual precipitations promotes the persistence of particles in the atmosphere. Metal-containing particles of the atmosphere may be directly retained by plant leaves or accumulate in soils after their deposition. To investigate date palm potential to uptake and transport metals, ex situ assays were performed in soil spiked with increasing concentrations of Cd, Cu and Cr. Results presented in Table 1 show great capacities of the plant to accumulate Cd. Bioaccumulation factor of Cd ranged from 1.07 to 1.72, suggesting metal transfer from soils treated with 10 and 50 mg kg^−1^ to the plant (Table 2). This ability to absorb metal may be related to its great TF against an important enrichment factor especially at low metal concentration (Table 2). Because metals may compete with each other, the abundance of a particular metal can disturb the uptake of others. For example, our results suggest that Cd can disturb Mn uptake by the plant (Table 3). Similarly, Dong et al. [54] indicated the existence of a negative correlation between Cd and Mn uptake in tomato plants exposed to Cd-stress. Such antagonism may restraint leaf photosynthesis and plant growth [55] observed in our case (data not shown). Non-essential metals may compete differently. For instance, Cr induced stress enhanced Mn accumulation in roots of the plant, while it decreased it in leaves (Table 3). In fact, previous reports showed that mobilization of micronutrients, especially Mn, into the rhizosphere is due to its acidification and complexation with organic acids (citrate) in various plant species [56]. Yet, citric acid with its strong affinity to form complexes with HM reduces the mobility of metals to the shoots. Furthermore, the obtained values of BCF of Cu in roots of date palm at low metal amount (1.37), as well as those of TF (0.9), suggested that plants accumulate and stabilize this metal within these compartments. However, increasing Cu stress reduces metal absorption and translocation, especially at 500 mg kg^−1^ Cu, suggesting that date palm regulated intracellular Cu levels by regulating Cu absorption.

### 4.2. Impact of Non-Essential Metal Uptake on Biochemical Integrative Biomarkers in Date Palm

The ability of organisms to acclimate metabolically to metal stress in the environment has interested researchers for years [57]. In this context, H_2_O_2_ accumulation in plant is a key regulator in numerous physiological processes [58]. The risk of oxidative damage by accumulating H_2_O_2_, naturally exists in plant cells where oxidant accumulation are more important in green cells [59]. For instance, in control leaves, H_2_O_2_ level was 1.5 time more important than in control roots of date palm plantlets (Table 3). Therefore, with no ability to produce ROS directly, the mechanism of Cd-induced oxidative stress is different from other forms of stress [60]. Cd caused accumulation of some oxidant. Indeed, H_2_O_2_ amount in roots of young date palm plants grown in 100 mg kg^−1^ of Cd increased by more than 4 times compared to control (Table 4). This may be due to the fact that Cd decreases protective enzymatic defense mechanism, especially SOD activity. This latter decreased significantly with increasing Cd stress in roots and leaves parts of date palm (Table 4). Similarly, Romero-Puertas et al. [61] showed Cu/Zn SOD downregulation in pea plants exposed to Cd stress. Moreover, Cd may also displace Fe from proteins and increase free Fe that is responsible for ROS generation [60]. With a positive correlation between tissues and metal concentration (*p* < 0.05), APX activity increased in parallel to H_2_O_2_ accumulation in cells (Table 4). In fact, various environmental stimuli, such as H_2_O_2,_ can modulate the expression of APX encoding genes [62]. Similarly, coffee cells increased APX activity at the lower Cd concentration [63]. At high metal amount, APX activity dropped down in roots. This may be due to glutathione (GSH) depletion and a subsequent reduction in the ascorbate–glutathione cycle [63]. In fact, the GSH reduction could be caused by PCs synthesis induction provoked by Cd^2+^ ions. Indeed, in the present research, *Pdpcs1* gene encoding PCs synthesizer enzyme using GSH, showed the highest induction in roots treated with 50 mg kg^−1^ Cd (Figure 2) were APX activity started to be reduced (Table 4). Furthermore, phenolic content of date palm cells was significantly affected by high Cd concentration (Table 4). With their antioxidant properties, phenolic compound accumulation in cells represents a key factor of induction of the defense mechanisms of plants through the phenylpropanoid pathway [64]. Significantly, higher accumulation of non-enzymatic oxidant scavengers was noted in leaves of date palm treated with Cd except for flavonoid content, which showed the highest induction in leaves of plants treated with 50 mg kg^−1^ Cd (Table 4). It is oxidized by peroxidase, and it acts especially in H_2_O_2_-scavenging [65].

As a highly mobile strong oxidizer, Cr in its hexavalent form may cause severe phytotoxic effect. It enhanced H_2_O_2_ and TBARS level in cells (Table 3). Cr^VI^ is catalytically more active than Cd and less active than Cu, and is able to generate ROS via Fenton reaction [66]. As shown by Ahemad [66], Cr^VI^ toxicity is related to its easy diffusion through the membrane cells, which leads to the production of free radicals. The relationship shown between Cr^VI^ accumulation and oxidant generation can be understood as a negative influence of increasing metal accumulation on redox balance. The increase in H_2_O_2_ could be explained by the enhancement of SOD activity that is in correlation with the respective Cr-accumulation in plant (Table 1 and Table 3). In fact, Fe-SOD was found to be the predominant form of Cr-induced SOD in stressed plants [67]. However, excess of Cr was shown to interact with essential nutrient like Mn (Table 3) and especially Fe [68]. It decreased the uptake and translocation of Fe ions, decreasing SOD activity including Fe-SOD enzyme activity. APX continued to overproduce in all tested Cr concentrations and especially at the highest concentration (Table 4). This plant response to Cr defended the potential of APX to gain oxidative damage. Similar findings were reported in other plant species, such as sorghum [67,69], suggesting that APX might have provided sufficient antioxidant defense against H_2_O_2_ generation. Cr salt also induced the biosynthesis of secondary metabolites especially polyphenol and flavonoids, particularly at 100 mg kg^−1^ of Cr (Table 4). Similar findings were shown in *Brassica juncea* exposed to Cr stress [70]. Moreover, Dubey et al. [71] reported that the most upregulated genes in response to Cr-stress are related to biosynthesis of secondary metabolites, especially flavonoid biosynthesis.

### 4.3. Essential Metal Uptake Effects on Biochemical Integrative Biomarkers in Date Palm

Cu is a transitional element and it is known to cause oxidative damage in cells [72]. Despite of its great accumulation in plant tissues, it was found that Cu was less active than Cd as a producer of oxidative stressor like H_2_O_2_. The fact that Cu^2+^ ions are used as cofactors by numerous proteins and are required by the ethylene receptor for proper signaling may explain in part this oxidative state of cells. Yet, SOD activity increased gradually, when metal concentration increased in the plant (Table 4). Cu ions are required in the chloroplast as cofactors for SOD producing Cu/Zn SODs under a control of microRNAs [73,74]. Similarly to the behavior of *Moso bamboo*, (as Cu-hyperaccumulator plant) under cupric stress [75], the present results indicated that *P. dactylifera* can induce the activity of essential components of plant enzymatic antioxidant system, to avoid oxidative damage induced by Cu toxicity. However, high Cu stress caused overproduction of H_2_O_2_, decreased enzymes activity, and damaged biomolecules, such as membrane lipids (Table 4). Plants grown in soil containing 100 mg kg^−1^ of Cu secreted polyphenols, flavonoid, and tannins as additional mechanisms of self-protection to reduce toxic effect of Cu^2+^ (Table 4). Polyphenols are known to be involved in ROS scavenging due to their chemical structure which also helps to bind with HMs, especially Fe and Cu [76]. In addition, flavonoids are shown to be produced in oxidative stress conditions, where transition metals ions, such as Fe and Cu, are involved. With their chelating properties and their ability to locate and neutralize ROS from cells, flavonoids have higher reducing capacity for Cu ions and act as ROS scavengers [38]. Similar increase in total flavonoids content was shown in the medicinal plant *Orthosiphon stamineus* exposed to Cu stress [77].

The biochemical responses described above, including integrative biomarkers for metallic stress, were further supplemented by the measure of the level of expression of selected genes.

### 4.4. Infra-Individual Expression Biomarkers for HMs Stress on Date Palm

Expression levels of six selected genes were monitored in date palm exposed to Cd, Cu, and Cr stress. Implication of metal chelators and transporters may be achieved by differential regulations at transcriptional and/or post-transcriptional levels. Only Cd at high concentration repressed expression of some genes (Figure 2), namely *Pdpcs1*, *Pdabcc* family, and *Pdmate5*. However, the highest *Pdpcs1* induction was observed in leaves of plant treated with 50 mg kg^−1^ Cd inducing the biosynthesis of PCS enzyme. Differently, Cobbett and Goldsbrough [22] have shown that in *Arabidopsis* PCS is constitutively expressed and post-translationally activated, especially by Cd^2+^. The synthesis of the small metal binding peptides (PCs) under control of PCS play a role in cellular homeostasis and trafficking of essential nutriment, particularly Cu and Zn [78,79]. They are also required for detoxification of toxic HMs, such as Cd. In addition, increasing metal amount adversely affect *Pdpcs* expression in hypocotyls of date palms exposed to Cd, as shown previously [30]. In different plant tissues, increasing Cr concentration increased gene expression and the highest induction factor of *Pdpcs1* was observed at high concentration (500 mg kg^−1^ Cr), especially in date palm leaves (Figure 2a). PCs bind HM ions to form high-molecular-weight (HMW) to be transported into the vacuoles across the tonoplast by the ABCC type transporters. A correlation between *Pdpcs1* and *Pdabcc-type* expression was found (Figure 2a,c) and calculated by Statistica Pairwise Correlation coefficient (*r*). It was found that at 10 and 50 mg kg^−1^ Cd, *r* obtained values were almost equal to +1 (0.954 and 0.990 respectively), suggesting a perfect positive linear relationship. Furthermore, our results suggested that Cu and Cr ions were more likely to be up taken and translocated into vacuoles than Cd ions, which is in accordance to metal concentrations in plant tissues (Table 1). This may be a part of the plant defense mechanisms. Furthermore, Cr plant resistance may be explained by the overexpression of *mt3* (Figure 2b). In sorghum Cr^VI^ tolerant variety, higher transcription rates of the MT3 were observed [69]. Others suggested that H_2_O_2_ produced under Cr stress acted as a signal to induce MT mRNA transcript. This is in accordance to our results which showed that H_2_O_2_ level in date palm treated with Cr and Cu increased with increasing metal amount (Table 3). Moreover, *Pdmt3* was more responsive under Cu stress than under Cd stress suggesting that this gene could be specifically involved in Cu detoxification similarly to other species [80]. Furthermore, MTs participate in controlling the concentration of ROS that would activate defense mechanisms, e.g., via the mitogen-activated protein kinase (MAPK) cascade [81]. The complex HM-MT is then translocated into vacuole by ABCC transporters. However, the P-type ATPases proteins transport a variety of cations across cell membranes. The HM-transporting (HMA) subfamily of P_1B_-ATPase contains different genes. HMAs involved in HMs uptake are known to be more selective than the other transporter proteins. HMAs gene belongs to the cation diffusion facilitator (CDF) protein family and is involved in Zn and Cd transport. Interest was focused on *hma2*, which respond differently to different metal stress (Figure 2d). In natural conditions, the expression of *Pdhma2* was significantly (*p* < 0.05) similar in the roots and shoots accordingly to wheat plants [82]. It increased significantly with increasing metal stress, even at high metal amount. *Pdhma2* relative expression factor reached 2.6 and 2.5 in roots, and leaves of 100 mg kg^−1^ Cd-treated plants, respectively. Studies in *Arabidopsis* showed the involvement of *Athma2* and *Athma4* in Zn/Cd translocation through xylem loading, as they act as pumps to efflux Zn/Cd out of cells suggesting a putative role of HMA2 in long-distance transportation. Yet, *Pdhma2* relative expression factor was much higher under Cr conditions and it was induced even in 50 mg kg^−1^ of Cr. However, no significant effect of Cr concentrations on gene expression was shown in plant leaves compared to root tissues (Figure 2d). The gene maintained high expression level during metal presence in tissues. This finding suggested high *hma2* sensitivity to Cr in date palm. In literature, no involvement of HMA2 in Cr uptake has been found yet. However, HMA5 encoding gene was upregulated by Cr^VI^ ions and was indispensably implicated in the regulatory network responsive to Cr stress in radish [83]. Similarly, Cr induced *Nramp6* expression by almost 3-fold in date palm roots and leaves (Figure 2e). The *Nramp6* was more expressed in regulating Fe homeostasis and metal transport. The target gene expression enhancement under Cr-stress may be explained by the fact that the forms of Cr^VI^ are reduced by Fe^2+^ uptake [81]. It was also induced in Cd stressed-plantlets, especially at low metal amount. Member 6 of Nramp family encoding gene was not much studied before. Yet, in rice, *Nramp1* was found to be highly expressed in roots and shoots of plants treated with Cd contrarily to *Nramp5* [84]. In addition, transporter encoding gene belongs to the family of citrate transporters (Multi antimicrobial extrusion protein MATE family protein); *Pdmate5* expression responded to metal stress. MATE transporters are localized in both vacuole and plasma membranes, and are involved in a wide range of biological processes in plants, such as transport of secondary metabolites especially flavonoids, alkaloids, and anthocyanidins, detoxification of HMs, Fe translocation and Al detoxification, and efflux of plant hormones, such as abscisic acid [76]. *mate* are expressed especially in response to Fe ions presences in cells. Thereby, MATE proteins participate in Fe-citrate efflux and are engaged to translocate Fe^2+^ from the roots to leaves [85]. In our study, *Pdmate5* transcription enhanced under stress except at high Cd level (Figure 2f). There is in correlation to flavonoids behavior under Cd stress. Similarly, MATE was found to be upregulated under Cr-stress in rice as a transporter of flavonoids [71]. Because flavonoid composition is different in each plant species, the transport activity of MATE transporters could be different. However, in rice, MATE proteins were proved to be involved in Cu resistance by secreting Cu-conjugates as secreting Al-citrate in response to Al-stress [86]. MATE were also upregulated under Cu stress in grapevine [87].

## 5. Conclusions

Facing to atmospheric emission by industries of Sfax region, the surrounding areas suffer from a metallic contamination. Focusing on the date palm as the perennial plant that was exposed to contamination for a long time, we demonstrated that Cd, Cu, and Cr levels in field collected plant leaves exceed the permissible limits. Nevertheless, plants continue to survive in such environment. The investigation for strategies for detoxification of HMs in the date palms, maintained in controlled conditions, made it possible to identify integrative biomarkers for each metal tested. Thus, non-specific biomarkers were monitored in plants exposed to Cd or Cu or Cr spiked soils. H_2_O_2_, TBARS, and CAT levels showed increase mainly in roots of Cd-exposed plantlets. However, SOD and APX activity and flavonoid production were stronger in response to excess Cu and Cr. As specific biomarkers, gene regulatory networks play crucial roles in Cu homeostasis and Cd or Cr detoxification. Cd stress induced *Pdpcs1* more than the *Pdmt3* gene. Cd also increased the expression of the genes *Pdhma2* and *PdNramp6,* which encoded proteins implied into translocation of metals to the leaves. This may explain the important TF of Cd in plantlets. Following exposure to Cu, marked increases in the expression of *Pdpcs1*, *Pdhma2* and *PdNramp6* were noticed. Exposure to Cr induced *Pdmt3* gene expression. Moreover, in the present study, we selected individual markers of HMs effects on date palm that include responses of the plant at a molecular level, and may also contribute to the design of genetic tools, to identify more efficient plants for phytoremediation.

## Figures and Tables

**Table 1:** Mean metals content in different compartments of site collected samples and ex-situ experiments.

								Doped Soils											
		Uncontaminated Site			Contaminated Site			Cd (mg kg^−1^)				Cu (mg kg^−1^)				Cr (mg kg^−1^)			
		Cd	Cu	Cr	Cd	Cu	Cr	0	10	50	100	0	50	100	500	0	50	100	500
**Metal Concentration (mg kg^−1^)**	Soil	0.3 (0.1)	5.4 (0.4)	0.1 (0.1)	0.9 (0.1)	7.5 (0.3)	28.0 (0.8)	0.7aA (0.02)	4.4aB (0.2)	26.3aC (1.1)	63.9aD (0.9)	5.68aA (0.02)	19.3aB (1.4)	33.4aC (2.5)	64.7aD (0.9)	0.05aA (0.02)	26.5aB (2.0)	71.8aC (1.6)	29.2aD (3.9)
	Roots	-	-	-	-	-	-	0.2bA (0.02)	4.5aAB (0.0)	16.0aBC (5.04)	27.1bC (5.1)	4.85aA (0.02)	17.2abB (2.9)	18.8bB (2.1)	19.6bB (1.9)	0.64bA (0.02)	9.8bB (0.6)	15.4bB (0.8)	25.8bC (0.2)
	Leaves	0.2 (0.0)	3.8 (0.7)	0.1 (0.8)	1.6 (0.7)	14.6 (2.8)	17.9 (1.9)	0.4cA (0.09)	2.1aA (1.5)	12.2bB (1.2)	15.3abB (3.7)	3.82bA (0.09)	9.3bB (1.2)	12.7bB (1.9)	13.8bB (0.7)	0.02aA (0.09)	7.2bB (0.3)	8.7bB (0.2)	14.2cC (2.5)
**Zn Concentration (mg kg^−1^)**	Soil		9.4 (0.2)			15.4 (0.7)		19.7aA (0.4)	34.9aB (0.0)	36.1aC (0.1)	41.2aD (0.3)	19.7aA (0.4)	23.91aA (1.8)	29.8aA (5.3)	32.82aA (2.8)	19.7aA (0.4)	25.1aB (0.1)	36.32aC (0.2)	29.85aC (0.6)
	Roots		-			-		17.50bA (0.6)	10.47bB (0.6)	8.4aAB (2.0)	6.6aAB (2.2)	17.5bA (0.6)	16.93bB (0.2)	11.14aC (0.3)	10.2aD (0.0)	17.5bA (0.6)	14.3bB (0.4)	9.2bC (3.0)	8.8bC (4.0)
	Leaves		14.0 (1.1)			36.0 (1.5)		29.3cA (0.9)	18.9aA (1.4)	15.5bA (0.7)	6.3bB (0.5)	29.3cA (0.9)	22.8abB (2.8)	10.87aC (0.2)	8.85aC (0.0)	29.3cA (0.9)	18.4bB (0.3)	11.81aC (4.0)	10.82aC (0.1)

Results shown as mean ± SD. Differences between groups are shown as results of two-way ANOVA post-hoc Tukey’s test, where small letters show differences between compartments (in rows) and major letters show differences between metal concentrations (in columns). Means not showing the same letter are statically different.

**Table 2:** Values of related metal accumulation factors. Results shown as mean ± SD.

	Cd (mg kg^−1^)				Cu (mg kg^−1^)				Cr (mg kg^−1^)			
	0	10	50	100	0	50	100	500	0	50	100	500
**BAF**	0.64 (0.0)	0.74 (0.2)	0.46 (0.1)	0.24 (0.1)	0.29 (0.0)	0.49 (0.0)	0.38 (0.1)	0.21 (0.1)	0.38 (0.0)	0.27 (0.0)	0.12 (0.0)	0.04 (0.0)
**EF**	0.15 (0.4)	4.94 (0.4)	4.48 (0.3)	5.87 (0.2)	0.64 (0.0)	1.28 (0.5)	1.99 (0.1)	1.39 (0.7)	5.55 (0.4)	0.08 (0.0)	0.1 (0.02)	0.3 (0.0)
**TF**	0.23 (0.)	1.02 (0.0)	0.60 (0.0)	0.42 (0.1)	1.77 (0.)	0.9 (0.2)	0.56 (0.0)	0.30 (0.0)	1.5 (0.0)	0.90 (0.1)	0.56 (0.0)	0.30 (0.0)
**BCF**	0.93 (0.1)	1.72 (0.0)	1.07 (0.0)	0.66 (0.1)	0.19 (0.1)	1.37 (0.1)	0.98 (0.3)	0.51 (0.0)	1.89 (0.1)	0.37 (0.0)	0.21 (0.0)	0.08 (0.0)

BAF: bioaccumulation factor; EF: enrichment factor; TF: translocation factor; BCF: bioconcentration factor.

**Table 3:** Mn content in different compartments of site collected samples and ex-situ experiments.

				Doped Soils											
		Uncontaminated Site	Contaminated Site	Cd (mg kg^−1^)					Cu (mg kg^−1^)			Cr (mg kg^−^^1^)			
				0	10	50	100	0	50	100	500	0	50	100	500
**Mn Concentration (mg kg^−1^)**	Soil	9.0 (0.4)	14.1 (1.8)	40.7aA (0.3)	42.2aB (0.3)	44.9aC (0.0)	44.9aC (0.1)	40.7aA (0.3)	41.8aB (1.5)	44.0aAB (0.0)	44.3aB (0.3)	40.7aA (0.3)	46.8aA (2.6)	43.6aA (1.9)	46.4aA (2.0)
	Roots	-	-	4.20bA (0.4)	6.2bB (0.3)	4.8bAB (1.0)	2.2bA (0.3)	4.2bA (0.4)	6.1bB (0.1)	8.4bC (0.7)	4.0bA (0.5)	4.2bA (0.4)	8.4bB (0.5)	7.8bC (0.2)	6.8bBC (1.1)
	Leaves	4.5 (0.9)	16.5 (1.3)	18.3cA (0.5)	12.3cB (0.5)	7.1bC (0.2)	5.1cD (0.3)	18.3cA (0.5)	11.2bB (0.3)	8.6bC (0.5)	5.6bD (0.5)	18.3cA (0.5)	12.3bB (0.4)	8.9bC (0.0)	7.1bD (0.2)
				**Pairwise Correlation coefficient (*r*)**											
**Metal concentration-Mn concentration**				0.914191		−0.611946		0.914191		−0.48363		0.914191		−0.33352	

Results shown as mean ± SD. Differences between groups are shown as results of one-way ANOVA post-hoc Tukey’s test where small letters show differences between compartments (in rows) and major letters show differences between metal concentrations (in columns). Means not showing the same letter are statically different. Pairwise correlation coefficient (*r*) was calculated by Statistica 10. Red value showed correlation.

**Table 4:** Monitoring of elements of oxidative and antioxidative state of date palm plantlets subjected to different Cd, Cu, and Cr concentrations.

		H_2_O_2_ µmol/g FW		TBARS nmol/g FW		CAT µmole H_2_O_2_/mg Protein		APX		SOD U/mg Protein		Polyphenol %		Flavonoid %		Tannins %	
		Root	Leaves	Root	Leaves	Root	Leaves	Root	Leaves	Root	Leaves	Root	Leaves	Root	Leaves	Root	Leaves
	0	156.1aA (4.4)	244.6bA (5.8)	44.9aA (2.9)	56.5bA (4.0)	1.6aA (0.0)	1.7aA (0.2)	8.7aA (0.2)	9.9bA (0.6)	444.1aA (6.2)	550.1bA (28.7)	100aA (0.00)	100aA (0.00)	100aA (0.00)	100aA (0.00)	100aA (0.00)	100aA (0.00)
**Cd (mg kg^−1^)**	10	270.4aB (1.3)	381.9bB (22.0)	62.5aB (4.7)	70.4bAB (0.7)	2.7aB (0.1)	3.5bB (0.0)	12.5aB (0.1)	13.8bB (0.2)	590.3aB (9.3)	612.3bB (8.5)	114.8aB (4.7)	153.2bB (3.8)	201.5aB (3.3)	201.1aB (6.1)	132.4aB (4.19)	113.8bB (3.4)
	50	568.2aC (12.2)	599.6bC (1.7)	94.1aC (4.1)	86.8aC (9.7)	1.5aA (0.0)	1.8bC (0.0)	12.7aB (0.2)	14.7bB (0.1)	540.0aB (13.0)	542.8aA (28.1)	134.6aC (1.4)	145.0aB (10.6)	186.3aB (9.8)	205.2aB (6.5)	109.5aA (5.8)	150.0bC (5.4)
	100	709.5aD (18.7)	598.5bC (6.5)	98.3aC (3.0)	82.2bBC (2.7)	0.9aC (0.0)	0.4bD (0.0)	6.1aB (0.1)	11.4bC (0.7)	310.6aC (10.0)	373.6bC (22.4)	72.0aD (4.3)	83.7bC (2.8)	85.4aC (5.9)	60.6bC (8.0)	55.8aC (4.2)	64.9bD (1.7)
**One-way and Two-way ANOVA main effects**																	
**Tissues**		0.000 *		0.000 *		0.000 *		0.000 *		0.000 *		0.000 *		0.000 *		0.000 *	
**Concentration**		0.000 *		0.626		0.000 *		0.000 *		0.000 *		0.000 *		0.520		0.000 *	
**Tissues x Concentration**		0.000 *		0.000 *		0.002 *		0.003 *		0.001 *		0.000 *		0.000 *		0.000 *	
**Cu (mg kg^−1^)**	0	156.1aA (4.4)	244.6bA (5.8)	44.9aA (2.9)	56.5bA (4.0)	1.6aA (0.0)	1.7aA (0.2)	8.7aA (0.2)	9.9bA (0.6)	444.1aA (6.2)	550.1bA (28.7)	100aA (0.00)	100aA (0.00)	100aA (0.00)	100aA (0.00)	100aA (0.00)	100aA (0.00)
	50	202.9aB (4.6)	248.4aA (20.9)	44.3aA (3.4)	63.5bAB (3.1)	2.0aB (0.1)	2.0aAB (0.1)	10.9aB (0.1)	11.8bB (0.2)	583.8aB (13.8)	633.5bB (17.7)	116.7aA (7.0)	127.2aB (3.8)	238.1aA (6.6)	247.8aB (1)	148.4aB (7)	151.8aB (6)
	100	319.4aC (17.8)	325.0aB (25.0)	53.5aA (2.31)	69.2bBC (3.17)	2.4aC (0.1)	2.4bBC (0.0)	12.5aBC (0.1)	13.7bC (0.2)	660.8aC (12.3)	717.1bC (9.5)	151.5aB (6.3)	185.2bC (12.5)	271.2aA (18.1)	184.8bC (11.4)	162.6aC (6.5)	179.8bC (2.8)
	500	429.1aD (20.2)	464.3aC (15.4)	79.5aB (7.5)	88.1aC (0.8)	2.3aBC (0.2)	2.8bC (0.1)	11.9aC (1.4)	14.1aC (0.1)	829.6aB (5.5)	776.0bC (11.)	100.8aA (12.8)	110.2aAB (7.68)	223.7aA (8.2)	94.5bA (12.9)	87aA (4.3)	119.7bD (6.2)
**One-way and Two-way ANOVA main effects**																	
**Tissues**		0.000 *		0.000 *		0.000 *		0.000 *		0.000 *		0.000 *		0.613		0.000 *	
**Concentration**		0.015 *		0.000 *		0.340 *		0.001 *		0.000 *		0.990		0.539		0.004 *	
**Tissues x Concentration**		0.352		0.175		0.780		0.453		0.350		0.363		0.648		0.034 *	
**Cr (mg kg^−1^)**	0	156.1aA (4.4)	244.6bA (5.8)	44.9aA (2.9)	56.5bA (4.0)	1.6aA (0.0)	1.7aA (0.2)	8.7aA (0.2)	9.9bA (0.6)	444.1aA (6.2)	550.1bA (28.7)	100aA (0.00)	100aA (0.00)	100aA (0.00)	100aA (0.00)	100aA (0.00)	100aA (0.00)
	50	183.9aA (18.1)	268.5bA (16.0)	45.1aA (1.9)	59.0bA (1.9)	2.1aAB (0.0)	1.8aAB (0.1)	11.0aB (0.1)	11.4bB (0.1)	503.5aB (7.6)	576.0bA (9.7)	120.2aB (1.2)	152.6bB (2.6)	152.5aB (3.6)	130.7bB (1.3)	169.6aB (3.5)	153.0bB (5.9)
	100	317.7aB (3.2)	347.9aB (32.7)	60.6aB (7.5)	72.6aB (2.4)	2.4aBC (0.3)	2.2aBC (0.1)	12.6aC (0.3)	12.2aB (0.2)	617.1aC (26.8)	684.5bB (11.1)	160.6aC (6.7)	217.8bC (0.4)	272.8aC (8.3)	210.2bC (8.9)	127.0aC (3.5)	181.3bC (6.2)
	500	589.4aC (11.7)	520.9bC (27.6)	83.0aC (2.7)	87.1aC (0.8)	2.7aC (0.2)	2.3aC (0.1)	14.5aD (0.4)	14.5aC (0.3)	730.7aD (11.6)	787bC (16.6)	130.1aD (1.0)	113.6bD (9.4)	199.00aA (4.1)	151.6bA (1.5)	103.5aA (3.3)	112.4aA (6.7)
**One-way and Two-way ANOVA main effects**																	
**Tissues**		0.000 *		0.000 *		0.000 *		0.000 *		0.000 *		0.000 *		0.000 *		0.000 *	
**Concentration**		0.229		0.005 *		0.065		0.154		0.001 *		0.342		0.519		0.177	
**Tissues x Concentration**		0.022 *		0.440		0.219		0.149		0.640		0.029 *		0.900		0.470	

Results shown as mean ± SD. Differences between groups are shown as results of one-way and two-way ANOVA post-hoc Tukey’s test where small letters show differences between tissues (in columns) and major letters show differences between concentrations (in rows). Means not showing the same letter are statically different. * level of significant (<0.05).

## Data Availability

Not applicable.
